# A Divergent Artiodactyl *MYADM*-like Repeat Is Associated with Erythrocyte Traits and Weight of Lamb Weaned in Domestic Sheep

**DOI:** 10.1371/journal.pone.0074700

**Published:** 2013-08-30

**Authors:** Michael V. Gonzalez, Michelle R. Mousel, David R. Herndon, Yu Jiang, Brian P. Dalrymple, James O. Reynolds, Wendell C. Johnson, Lynn M. Herrmann-Hoesing, Stephen N. White

**Affiliations:** 1 Animal Disease Research Unit, Agricultural Research Service, U.S. Department of Agriculture, Pullman, Washington, United States of America; 2 Department of Veterinary Microbiology and Pathology, Washington State University, Pullman, Washington, United States of America; 3 U.S. Sheep Experiment Station, Agricultural Research Service, U.S. Department of Agriculture, Dubois, Idaho, United States of America; 4 CSIRO Animal, Food and Health Sciences, St. Lucia, Australia; University of Queensland, Australia

## Abstract

A genome-wide association study (GWAS) was performed to investigate seven red blood cell (RBC) phenotypes in over 500 domestic sheep *(Ovis aries)* from three breeds (Columbia, Polypay, and Rambouillet). A single nucleotide polymorphism (SNP) showed genome-wide significant association with increased mean corpuscular hemoglobin concentration (MCHC, *P* = 6.2×10^−14^) and genome-wide suggestive association with decreased mean corpuscular volume (MCV, *P* = 2.5×10^−6^). The ovine HapMap project found the same genomic region and the same peak SNP has been under extreme historical selective pressure, demonstrating the importance of this region for survival, reproduction, and/or artificially selected traits. We observed a large (>50 kb) variant haplotype sequence containing a full-length divergent artiodactyl *MYADM*-like repeat in strong linkage disequilibrium with the associated SNP. *MYADM* gene family members play roles in membrane organization and formation in myeloid cells. However, to our knowledge, no member of the *MYADM* gene family has been identified in development of morphologically variant RBCs. The specific RBC differences may be indicative of alterations in morphology. Additionally, erythrocytes with altered morphological structure often exhibit increased structural fragility, leading to increased RBC turnover and energy expenditure. The divergent artiodactyl *MYADM*-like repeat was also associated with increased ewe lifetime kilograms of lamb weaned (*P* = 2×10^−4^). This suggests selection for normal RBCs might increase lamb weights, although further validation is required before implementation in marker-assisted selection. These results provide clues to explain the strong selection on the artiodactyl *MYADM*-like repeat locus in sheep, and suggest *MYADM* family members may be important for RBC morphology in other mammals.

## Introduction

Blood volume is 30–50% red blood cells (RBCs) which transport oxygen and carbon dioxide. Hematological measures are often used as a means of diagnosing clinical disorders and as an overall measure of health. However, genetic determinants affecting the expression of erythrocyte phenotypes are poorly understood, especially in domestic sheep and other livestock species [1], [2]. It has been estimated that roughly 40 – 90% of erythrocyte traits are heritable and due to genetic differences in the population, highlighting the importance of identifying as many of the remaining unknown mutations as possible [3]–[6].

Genetic disorders affecting erythrocyte morphology often have harmful effects on health, including increased RBC fragility, high cellular turnover, and inefficient trafficking of oxygen to the body’s tissues. Roughly 100,000 Americans are stricken with sickle cell anemia, while another 1 in 2,000 suffers the effects of hereditary spherocytosis and elliptocytosis [7]. Some of the underlying genetic mutations that impart altered morphology of RBCs have been identified in genes expressing proteins integral to the structure of the RBC membrane [8], [9]. However, there remain a number of inherited RBC morphological phenotypes in humans and other mammals in which the mutations or specific genes have not been identified [10].

Since the sheep’s domestication approximately 11,000 years ago, there has been genetic selection to maximize production traits such as meat, wool, and milk [11], [12]. The result of this domestication is the existence of a phenotypically diverse collection of breeds, which serve a variety of needs both on a global and domestic scale. Bottleneck events associated with breed formation may have allowed previously rare variants to achieve much higher frequency in some breeds. Additionally, erythrocyte production and quality are under a number of environmental and genetic influences [3]. It is well established that the effects of altitude can have wide-reaching influences on certain RBC indices [13], [14]. As foragers, sheep must adapt and cope with large changes in altitude particularly in U.S. Western rangeland production systems. The ability to handle these large changes in altitude may help the sheep survive and maximize the particular production values for which it was selected.

Recent advances in genotyping technology have made possible the identification of genes that play a role in the expression of many complex traits, such as erythrocyte traits. High density arrays able to interrogate over 50,000 single nucleotide polymorphisms (SNPs) have been released that allow the examination of genome-wide variants related to any number of measured phenotypes [12]. These genome-wide association studies (GWAS) rely on correlated inheritance of nearby alleles (haplotypes), known as linkage disequilibrium (LD). As a result, causal mutations are inherited along with linked genotyped markers in association with the particular phenotype under study.

In this study, a GWAS strategy was used to identify genomic regions harboring genetic variants important for seven RBC traits in domestic sheep from three important U.S. breeds. Additionally, a number of production traits were analyzed for association with a divergent artiodactyl *MYADM-*like repeat to address potential loss of production through increased energy costs of replacing fragile RBCs.

## Materials and Methods

### Ethics Statement

All animal care and handling procedures were reviewed and approved by the Washington State University Institutional Animal Care and Use Committee (Permit Number: 3171) and/or by the U.S. Sheep Experiment Station Animal Care and Use Committee (Protocol Numbers: 04-14, 10-07). All efforts were made to minimize any discomfort during blood collection.

### Populations and Phenotypes

#### Red Blood Cell Analysis

Whole blood was collected by jugular venipuncture from ewes of Columbia (N = 145), Polypay (N = 438), and Rambouillet (N = 414) breeds, aged 1–5 years, from the U.S. Sheep Experiment Station in Dubois, Idaho into EDTA-coated vacutainer tubes as previously described [15]. These animals were managed similarly but bred separately in purebred groups. Complete blood counts were performed on a subset of 521 sheep from the initial population described above including Columbia (N =  67), Polypay (N =  202), and Rambouillet (N =  252) sheep. Complete blood cell counts were performed by Phoenix Labs, Inc. (Everett, Washington, USA) roughly 24 hours from the time of collection. Seven RBC phenotypes were investigated. Four of these phenotypes were measured directly: red blood cell number (RBC, measured in M/µl, reference range: 4.0–12.0 M/µl), hemoglobin (HGB, measured in g/dl, reference range: 9.0–15.0 g/dl), hermatocrit (HCT, measured in %, reference range: 27.0–45.0%), and platelets (measured in K/µl, reference range: 250–750 K/µl). Three derived traits were also included in the analyses: mean corpuscular volume (MCV, measured in fL, reference range: 28.0–40.0 fL), mean corpuscular hemoglobin (MCH, measured in pg, reference range: 8.0–12.0 pg), and mean corpuscular hemoglobin concentration (MCHC, measured in %, reference range: 31.0–35.0%). All reference values were provided by Phoenix Labs, Inc. (Everett, Washington, USA).

#### Production Trait Phenotypes

Production and growth data were collected on ewes and lambs at the US Sheep Experiment Station as previously described [16]–[18]. In addition to the discovery population of 521 sheep from the RBC GWAS, 841 additional sheep, consisting of Columbia, Polypay, and Rambouillet breeds, were included in the production trait analysis. Of these, a total of 347 sheep, including 43 Columbia, 218 Polypay, and 145 Rambouillets were from a 2008 Idaho cohort. An additional 351 were included from an earlier cohort sampled in 2004 that did not include any overlapping animals [16]. Therefore, 1,362 sheep were used for production trait analyses. The production traits investigated included: ewe birth weight, ewe 120-day adjusted weaning weight, ewe third year mature weight, ewe fourth year mature weight, ewe third year udder score, ewe fourth year udder score, lifetime number of lambs born, lifetime number of lambs born alive, lifetime number of lambs born dead, lifetime kilograms of lamb born, and lifetime kilograms of lamb weaned. Udder score is a subjective measure of udder condition ranging from 1–12 with a score of 2 being ideal. Increased scores indicate less healthy udder condition. Udder score measurements are recorded yearly at approximately 160 days after parturition.

#### Identification of Copy Number Variants

Copy number variants (CNV) were identified based on the OARv3.1 genome assembly. BWA [19] was used to align paired end sequence (101 bp reads) with default parameters onto unmasked OARv3.1. The maximum edit distance was automatically chosen for mapped reads with >95% sequence similarity with the reference genome. Approximately 95% of reads could be mapped onto the reference OARv3.1 genome. Then we focused on the *MYADM*-like repeat region on ovine chromosome 18. The WSSD based CNV calling pipeline [20] was adjusted by using longer alignment reads and small window size to make depth calling more sensitive. The aligned read numbers were counted around the *MYADM*-like repeat region within 200 bp sliding windows and 100 bp slide steps using a perl script. All gapped and repeat containing 200 bp windows were filtered out. CNV calls were initially selected if five out of seven or more sequential 200 bp overlapping windows had read depth values that differed significantly from the average (duplications > mean + 2 x STDEV). If five out of seven or more sequential 200 bp overlapping windows had more than 0.5 average fold depth with more than 4 animals from the re-sequenced domestic animals, and a low depth (<0.1 average depth) was observed in some other animals, these regions were defined as a deletion or alternate region with <95% identity with reference sequence.

#### Partial Definition of Alternate Allele Sequence

Two complementary methods were used to refine the position of a divergent artiodactyl *MYADM*-like repeat region and determine the sequence of the alternate allele. The first took advantage of the reference sheep genome OARv3.1, and the second utilized a de novo assembly method. All genomic sequences used were downloaded from the Genome sequence comparison of various sheep breeds project (BioProject: PRJNA160933).

**Figure 1:**
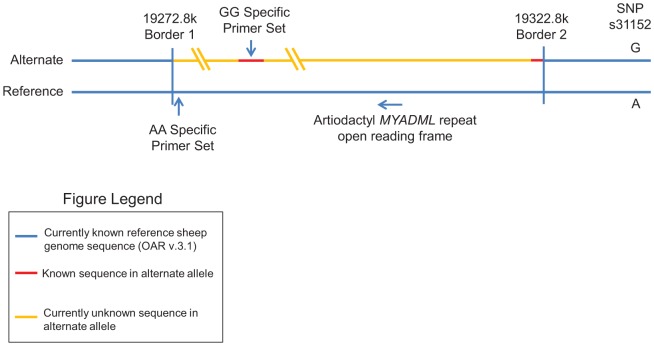
Artiodactyl *MYADM*-like Repeat Region of Alternate Alleles on Chromosome 18. A visual representation of what is known of the divergent artiodactyl *MYADM*-like repeat region of alternate alleles. The positions of allele-specific primer sets for each homozygote are specified.

In the first method, a ∼50 kb sequence region had very few reads mapped in a number of animals (from Oar18 19272.8 kb to 19322.8 kb, with a small distance, 20 kb to SNP s31152), which was defined as a deletion/alternate region (Fig. 1). Based on the junction reads around 19322.8 kb, it was determined that this ∼50 kb sequence region was not a deletion region, but that an alternative sequence segmental duplication exists in these animals (Fig. 1).

The second method to determine the sequence of the alternate allele relied on de novo assembly of additional animals. Briefly, genomic sequences from the *Ovis aries* diversity study (PRJNA160933, record SRR501852, MER454) were downloaded from the sequence read archive at NCBI and parsed into forward and reverse fastq files using the SRAToolkit. In order to reduce the complexity of the assembly and target the *MYADM*-like region of interest, sequences were mapped to the *Ovis aries* OAR3.1 reference genome sequence using Bowtie 2 [21]. Mapping was performed sequentially against two different indexes. The first index was comprised of all OAR3.1 chromosomes except chromosome 18. The second index contained chromosome 18 sequence following masking of 25 kb on either side of the area of interest. Non-mapped paired reads were imported into CLC genomics workbench (CLC bio) and assembled *de novo* with default parameters. Contigs > 2 kb were used for further analysis.

### Genotyping Methods

#### Genotyping with OvineSNP50 Infinium BeadChip

Genotyping was performed as described previously [15]. Briefly, DNA was isolated using the Invitrogen GeneCatcher™ gDNA 3-10 ml Blood Kit per manufacturers’ instructions (Life Technologies, Carlsbad, CA). Genotyping services were provided by Geneseek Inc. (Lincoln, NE) using the OvineSNP50 Infinium BeadChip (Illumina Inc., San Diego, CA) with a set of 54,977 SNP designed by the International Sheep Genome Consortium [12].

#### Genotyping Additional Animals at s31152 for Expanded Production Trait Population

Additional sheep from the expanded data set were genotyped at s31152 with TaqMan® Genotyping Assays. TaqMan assays were performed according to manufacturer’s instructions (Applied Biosystems, Foster City, CA) using the following primers and probes: Amplification Primer 1 (CCGGTTCATCCTTGAGAAACTCTT), Amplification Primer 2 (CCCCACAGATGTGCTAATGGT) and Probe 1 (CCTCCTCTTCTTCAACC) and Probe 2 (CTCCTCCTCTTCAACC). VIC and FAM fluorescent tags were used on the probes, respectively.

#### Genotyping of Divergent Artiodactyl MYADM-like Repeat with Allele-Specific Primer Sets

Allele-specific primer sets included 5′ TGCAAGACTGGTTCACCCCAGAT and 5′ CCGGCTTTATGTCAGTGATACGTG to amplify a 330 bp region of chromosome 18 containing the *MYADM*-like allele represented by the reference sheep genome assembly 3.1. Similarly, 5′ TAGAAGTACTCCTACCTGAACGA and 5′ CCAAACAAATCCCTGAGCCATTTC were used to amplify a 719 bp region of the divergent artiodactyl *MYADM*-like repeat. All reactions were performed using Taqman® Universal PCR master mix per manufacturer’s instructions (Applied Biosystems, Carlsbad, CA). All PCR reactions began with an initial denaturation step of 95°C for 10 minutes, followed by 40 cycles of 95°C for 30 seconds, ­­­­64°C for 30 seconds, and 68°C for 1 minute. PCR products were visualized by gel electrophoresis. For genotypes that were discordant with observed genotypes at the discovery SNP (s31152), the above procedure was repeated once to minimize the probability of inaccurate genotypes due to PCR failure.

### Statistical Analysis

#### Association Analysis with Red Blood Cell phenotypes

**Figure 2:**
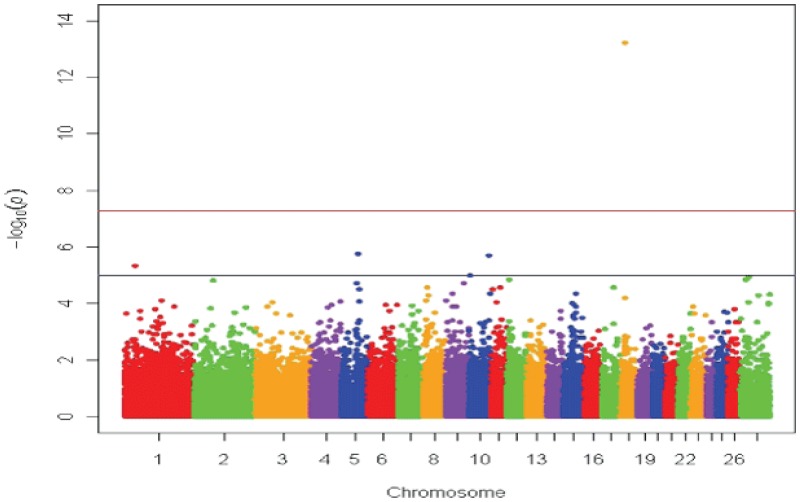
Manhattan plot for Mean Corpuscular Hemoglobin Concentration (MCHC) GWAS. The Manhattan plot shows nominal *P*-values from association with mean corpuscular hemoglobin concentration (MCHC) of erythrocytes by chromosomal position. Representative data from the all-breeds, genotypic mode of inheritance analysis is shown. The top red line shows a genome-wide significance threshold defined by nominal *P*-values of 1×10^−6^, which is *P* = 0.05/50,000. The lower blue line shows a genome-wide suggestive significance threshold defined by 1×10^−5^.

**Figure 3:**
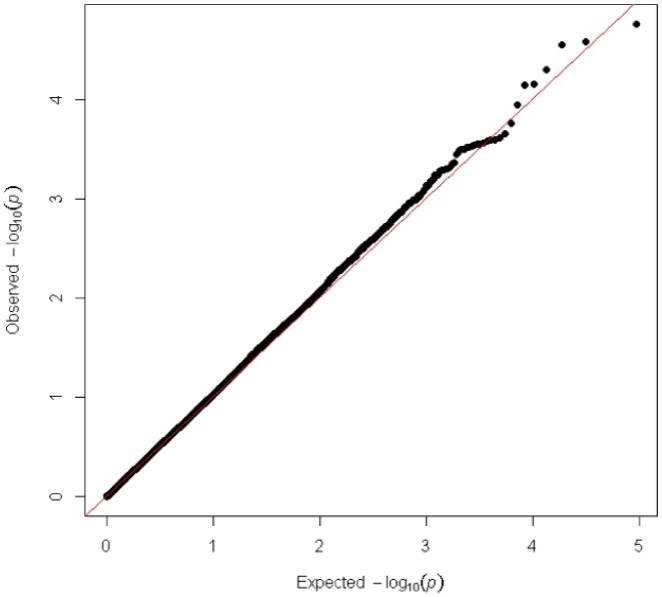
Quantile-Quantile plot for MCHC conditioned on Genome-Wide Significant and Suggestive SNPs. Quantile-quantile plot from association with MCHC, where the red line shows the expected distribution. Representative data from the all-breeds, genotypic mode of inheritance analysis is shown with the top 15 associated SNPs (by nominal *P*-value) are accounted for as fixed genotypic covariates in the statistical model. The results show very little evidence of population stratification once the top SNPs are accounted for in the model.

Red blood cell phenotypes were analyzed using analytic model methods as previously described [15]. Briefly, multidimensional scaling (MDS) and population concordance screening clusters were adopted from a previous study on the same animals [15]. Linear regression models in the PLINK software package (v. 1.06) [22] were used to account for breed and pairwise population concordance clusters previously described [15], [23], for animal age in years as a covariate, and for the SNP minor allele [15]. For all analyses, PLINK screening criteria included missingness by individual (individuals missing > 10% genotyping calls), missingness by marker (SNP markers missing >3% genotyping calls), minor allele frequency (>2%), and Hardy-Weinberg equilibrium (0.000001, which is *P* = 0.05 Bonferroni-corrected for 50,000 SNP tests). Genome-wide significance was defined by a nominal P≤5×10^−8^ [3], [23], [24]. Genome-wide suggestive results were defined by nominal *P*-values < 1×10^−5^ [3], [23], [24]. Association models included additive allelic, genotypic 2 degree-of-freedom, dominant, or recessive modes of inheritance to determine best fit. Visualization of association data in Manhattan and quantile-quantile plots was performed using a script provided by Dr. Stephen Turner (http://gettinggeneticsdone.blogspot.com/2011/04/annotated-manhattan-plots-and-qq-plots.html, viewed on 02-05-13) using the R platform (Fig. 2, 3, S1) [25]. SAS 9.2 (SAS Institute, Cary, NC) was used to run similar statistical models to those used with the PLINK software package in order to obtain the largest adjusted genotypic mean differences for each of the RBC phenotypes tested as a measure of effect size (Table 1, S6-11). Additionally, the top 15 SNPs reaching the genome-wide suggestive threshold were accounted for as fixed genotypic covariates in the all-breed, genotypic mode of inheritance MCHC analysis to show that very little evidence of population stratification exists (Fig. 3).

**Table 1:** Genomic regions associated with Mean Corpuscular Hemoglobin Concentration (MCHC).

SNP	*Chr*	*Position (bp)*	*Best fitting model*	*Nominal P-value*	*Effect Size*	*Other Significant Phenotypes*	*Genes within 100 kb on either side*
**s31152**	**18**	**19,342,316**	**genotypic**	**6.2×10^−14^**	**2.262**	**MCV**	***MYADM-like***
OAR11_22304711	11	21,548,639	allelic	3.5×10^−7^	1.125	None	*NXN, ABR, TIMM22*
OAR5_71365318	5	64,819,888	dominant	2.3×10^−7^	0.728	None	None
OAR10_87392185	10	80,078,689	allelic	9.5×10^−7^	0.873	None	None
OAR6_20258155	6	17,350,884	dominant	4.9×10^−7^	0.591	None	*LEF1, HADH, CYP2U1, SGMS2*
OAR4_124606219	4	116,753,479	allelic	6.2×10^−6^	0.822	None	*CNPY1, RBM33*
OAR6_74631720	6	68,106,616	allelic	6.6×10^−6^	1.137	None	None
s01540	18	18,935,512	allelic	6.9×10^−6^	0761	None	*MYADM-like*
OAR11_43352535	11	40,856,241	allelic	8.0×10^−6^	0.820	None	*KRT40, KRTAP1-4, KRTAP2-4, KRTAP4-7L, KRTAP4-8, KRTAP4-9, KRTAP4-12, KRTAP9-4, KRTAP9-7, KRTAP9-1, KRTAP3-3, KRTAP3-3L, KRTAP3-1L, KRTAP3-1, B2DL, B2AL, KRTAP1-1, B2CL, IIIA3L, KRTAPL, KRTAP4-12, KRTAP4-1, KRTAP4-7, KRTAP9-2L*
s28461	16	68,588,686	allelic	8.6×10^−6^	1.171	None	None
OARX_27576676	27	21,281,197	allelic	9.4×10^−6^	0.723	None	*APOO, KLHL15, CXHXorf58*
OAR3_22724265	3	21,102,941	dominant	5.2×10^−6^	0.570	None	None
OAR10_68313527	10	66,087,045	dominant	8.1×10^−6^	0.654	None	*MIR17HG, MIR18A, MIR19B1, MIR92A1, MIR17, MIR19A, MIR20A*
OAR1_43083702	1	41,602,942	genotypic	4.7×10^−6^	0.787	None	*PDE4B*
OAR10_4470416	10	6,292,394	genotypic	9.9×10^−6^	1.084	None	None

#### Marker-Production trait Associations

Production trait association testing was performed using the mixed model procedure of SAS 9.2 (SAS Inst. Inc., Cary, NC) with models as defined previously [18]. The models used to test all traits were age-adjusted models that included the trait of interest as the dependent variable. Birth year, breed, and marker genotype were included in the model as fixed effects for the following traits: ewe birth weight, ewe 120-day adjusted weaning weight, ewe third year mature weight, ewe fourth year mature weight, ewe third year udder score, and ewe fourth year udder score. Age at last lambing was included as an additional fixed effect for the following traits: lifetime number of lambs born, lifetime number of lambs born alive, lifetime number of lambs born dead, lifetime kilograms of lambs born, and lifetime kilograms of lamb weaned. Sire was included in the model as a random effect nested within sire breed for all traits considered. The *P*-values reported reflect single-locus Tukey-Kramer adjustment for multiple comparisons between genotypes.

## Results

### Genome-wide Association with Red Blood Cell Phenotypes

From the original set of 521 sheep, missing genotype and phenotype data eliminated 13 animals from additional analysis. The remaining animals included Columbia (N = 67), Polypay (N = 195), and Rambouillet (N = 247). After screening for genotyping call rate by individual and SNP, minor allele frequency, and Hardy-Weinberg equilibrium, the number of SNPs remaining for the RBC analysis was 47,563.

The RBC GWAS used seven erythrocyte phenotypes, including four directly measured parameters: RBC, HGB, HCT, and platelets and three derived traits: MCV, MCH, and MCHC. A total of 59 SNPs were associated with at least one of the seven RBC phenotypes investigated (Tables 1, S1-6). Additionally, 5 SNPs achieved genome-wide significance (Tables 1, S4-6), including s31152 (Fig. 2, Tables 1, S1, S5). Upon further investigation, the minor allele at s31152 (A) was associated with significantly increased MCHC (outside clinically normal range for sheep) and lowered MCV, while the major allele (G) exhibited normal values for the same measures (Table S7). Additionally, the Ovine Hapmap SNP s31152 data had reference allele frequencies similar to the U.S. breeds reported here (Table S8-10). The other four SNPs to reach the genome-wide significance threshold were OAR1 192908082, s19887, s63011, and s48861; they were found to be highly associated with a number of RBC phenotypes including MCH, MCV, and platelet count (Tables S4-6).

### Identification of a Divergent Artiodactyl *MYADM*-like Repeat

The SNP s31152 maps to chromosome 18 in the reference sheep genome sequence, version 3.1, containing a large tandem array of around 40 genes (including probable pseudogenes) encoding MYADM-like proteins (hereafter, *MYADM*-like; http://www.livestockgenomics.csiro.au/cgi-bin/gbrowse/oarv3.1/, viewed on 04-08-13). Sequence read depth in the re-sequenced sheep identified a region near s31152 in which sequence reads did not map to the reference assembly. This region covered reference positions 19272.8-19322.8 kb and included the complete open reading frame of exactly one *MYADM*-like repeat (Fig. 1). To test for the presence of reference sequence, we used ∼19 evenly spaced primer sets (Table S11). While they all amplified in s31152 AA homozygotes, all failed to amplify in s31152 GG homozygotes (Table S11). Sequence data assembled by multiple methods with GG homozygotes identified partial sequence of a highly divergent (<95% sequence identity) artiodactyl *MYADM*-like repeat allele (GenBank accessions KC914877 and KC914878), apparently inserted in a similar genomic location (between OARv3.1 chromosome 18 positions 19272.8-19322.8 kb, Fig. 1). Region-specific PCR primers were designed from the partial sequence of this alternate allele (Fig. 1, Table S11). Genotypes obtained using region-specific PCR to track the divergent artiodactyl *MYADM*-like repeat showed strong linkage disequilibrium with SNP s31152 (D’ = 0.992, r^2^ = 0.97) in the expanded production trait animal set (Table 2). The divergent artiodactyl *MYADM*-like repeat also accounted for nearly the entire GWAS association peak, with similar *P*-values for MCHC, MCV, and HCT (Table 3, S12). Additionally, tracking of the divergent artiodactyl *MYADM*-like repeat displayed similar *P*-values when used in the production trait analyses (Table 4, S12). In the expanded production trait population 55 reactions (2.0% of the total) either failed or were discordant among the genotyping calls made between the two methods.

**Table 2:** Genotypic Frequency of Divergent Artiodactyl *MYADM-*like Repeat in Expanded Production Trait Population.

	Columbia	Polypay	Rambouillet
Ref Homozygote	0	54	4
Ref/Alt Heterozygote	13	259	86
Alt Homozygote	226	224	438

**Table 3:** Significant Phenotypic Values for Divergent Artiodactyl *MYADM-*like Repeat by Genotype.

	Ref Homozygote^1^	Ref/Alt Heterozygote^1^	Alt Homozygote^1^	Nominal P-value
MCHC (%)	36.1	34.3	33.9	1.1×10^−13^
MCV (fL)	32.3	33.6	34.3	6.2×10^−6^
HCT (%)	31.3	33.4	33.8	0.0022

1 Genotypes refer to Reference and Alternate alleles of the divergent artiodactyl *MYADM*-like repeat.

**Table 4:** Divergent Artiodactyl *MYADM-*like Repeat genotype contrasts for lifetime kilograms of lamb weaned (LkgLW) and udder condition at fourth year (UDDER).

	Ref Homozygote^1^	Ref/Alt Heterozygote	Alt Homozygote	P-value^2^
LkgLW (in kg)	-	225.6	241.1	0.0002
UDDER	-	2.13	2.50	0.024

1 There were few Reference allele homozygotes, which were never significantly different (P<0.05) from either of the other genotypes for these traits.

2 P-values reflect comparisons between heterozygotes and Alternate allele homozygotes only.

### Production Trait Associations

An expanded population of 1,362 sheep was used in the production trait analysis, but 41 animals were eliminated from further analysis due to missing production trait data or genotypes. Genotypes from both allele-specific PCR for the divergent artiodactyl *MYADM-*like repeat and s31152 gave highly similar results for all production trait associations (Tables 2, S10).

The divergent artiodactyl *MYADM*-like repeat was segregating in the expanded production trait population in all breeds tested. The reference gene, containing the 50 kb sequence in OARv3.1, was present in Columbia (2.2%), Polypay (33.1%), and Rambouillet (9.8%) sheep in differing frequencies (Table 2). Inheritance of the divergent artiodactyl *MYADM*-like repeat was associated with lifetime kilograms of lamb weaned (*P* = 2×10^−4^) and ewe fourth year udder score (*P* = 0.024) (Table 4). Ewes homozygous for the divergent artiodactyl *MYADM*-like repeat allele had increased lifetime kilograms of lamb weaned compared to heterozygous ewes. Ewes heterozygous at the divergent artiodactyl *MYADM*-like repeat had a healthier udder score at 4 years of age than the alternate allele homozygote (Table 4). The other production traits analyzed were not significantly associated with the divergent artiodactyl *MYADM*-like repeat genotype (*P*>0.05).

## Discussion

With the advancement of genotyping technologies, GWAS have become an increasingly appealing option to identify genes underlying complex traits [26]. Domestic animal GWAS have focused on a variety of phenotypes, including economically important traits such as milk production, meat quality, and body composition [27]–[29]. In this analysis of genome wide association data from 509 domestic sheep comprising three U.S. breeds, we identified 59 SNPs that showed genome-wide suggestive association with at least one of seven hematological parameters. Four of these traits were measured directly: RBC, HGB, HCT, and Platelets. In addition, we also tested three derived red cell measures: MCV, MCH, and MCHC (Tables 1, S1-6). Of these 59 SNPs, only 5 achieved genome-wide significance (Tables 1, S4-6), and only these will be discussed further. Efforts to replicate these associations in additional populations are currently ongoing.

The locus most highly associated with MCHC (*P* = 6.2×10^−14^), was SNP s31152 (Table 1) that was also associated with decreased MCV (*P* = 2.5×10^−6^) (Table S5). s31152 was the only SNP that achieved genome-wide significance in this genetic region on chromosome 18 due to the density of SNPs interrogated by the Ovine SNP50 beadchip. Due to the multiple *MYADM*-like repeats in the region, the designers did not put a normal density of SNPs in this genetic location, leaving an approximate gap from positions 19180231-19342316 in the OARv.3.1. In addition, domestic sheep have a relatively short LD length compared to other mammals [12], [15], which can reduce the number of SNPs in each peak. Mapping the re-sequencing project reads to the reference genome identified a long (>50 kb) non-mappable sequence that could have been a deletion in this genetic region. However, further analysis found that this was not a simple deletion but rather the alternate allele likely containing a highly divergent sequence. This was subsequently confirmed in GG homozygotes using allele-specific PCR. The discovered alternate allele was found to contain an entire open reading frame of a *MYADM* homolog. This divergent artiodactyl *MYADM*-like alternate allele covers positions 19272.8-19322.8 Mb on ovine chromosome 18 in the OARv3.1 reference sheep genome assembly [11]. Partial sequence for the alternate allele of the divergent artiodactyl *MYADM*-like repeat region suggests it contains highly divergent sequence not included in OARv3.1. It is important to note that there is much that is not known of this genetic region, including the full sequence that is apparently inserted at positions 19272.8-19322.8 Mb. Figure 1 depicts the best representation of the *MYADM*-like genetic region given existing data and knowledge to this point. Preliminary sequence analysis suggests that there may be a large amount of diverged sequence between the two haplotypes, and elucidating evolutionary origins will require more complete sequence of the alternate haplotype. Efforts to elucidate the full sequence contained in the divergent artiodactyl *MYADM*-like repeat region and investigate the evolutionary origins are ongoing. Allele-specific PCR genotyping showed the divergent artiodactyl *MYADM*-like repeat to be in extremely strong LD with s31152 (D’ = 0.992, r^2^ = 0.97), and genotypes showed highly similar association with all traits (Tables 3, S7).

Increased MCHC and decreased MCV in RBCs may be indicative of changes in the morphological structure of RBCs in sheep. It is important to note that these RBC phenotypes are interconnected to a certain extent. Four of the phenotypes investigated were measured directly including RBC, HGB, HCT, and platelets. In addition, three RBC derived traits were also investigated which included: MCV, MCH, and MCHC. Although MCV, MCH, and MCHC are derived from the four measured RBC phenotypes, they were included because they are commonly used in the differential diagnosis of certain types of anemia. This means that a lowered cell volume (MCV), for example, would cause an increased hemoglobin concentration per cell (MCHC). The association of s31152 with MCHC, but not the directly measured RBC phenotypes, implies that the alternate *MYADM*-like repeat allele is associated with a concurrent increase in HCT but drop in HGB. However, the association peak for MCHC was approximately 8 orders of magnitude more significant than MCV, indicating that MCHC was affected in these RBC’s to a greater degree than can be explained by the lowered MCV values. These phenotypic changes are often associated with forms of anemia such as sickling or spherocytosis [30], [31]. Normally, RBCs have a characteristic biconcave disc shape. However, known mutations in structural proteins of the RBC plasma membrane have been shown to cause the formation of spherical or elliptical erythrocytes in circulation [8], [32].

The *MYADM* gene, the nearest homolog to the divergent artiodactyl *MYADM-*like repeat in the alternate allele sequence, is a member of the myelin and lymphocyte (MAL) protein family containing 8 trans-membrane domains in the form of 2 MARVEL domains [33], [34]. The MAL protein family has been implicated in the apposition of cellular constituents at the plasma membrane in a number of tissues including spleen, prostate, testis, ovary, small intestine, colon, peripheral blood leukocyte, heart, brain, placenta, lung, liver, skeletal muscle, kidney, and pancreas [35]. Additionally, the MYADM protein is expressed in a number of cell lines including U937, HepG2, PC3, HeLa, Jurkat, ECV304, COS-7 and MDCK [34]. The *MYADM* gene family appears to be expanded in a number of artiodactyls including cow, pig, and goat relative to other mammals investigated 11,[36], [37]. *MYADM* gene products have been implicated in the proper membrane formation and organization in cells of the myeloid lineage [34], [38]. The gene products from the *MYADM* family are also widely expressed in a number of cell lines, including up-regulation in pluripotent stem cells destined to complete erythropoiesis [39]. However, to our knowledge, this is the first report of a *MYADM* gene family member associated with RBC abnormalities in any mammalian system.

Altering the structural morphology of the RBC causes decreased osmotic fragility and increased membrane rigidity in vivo [40], [41]. The increased turnover rate and increased structural rigidity of affected RBCs would suggest an increased amount of energy allocated for the production and replenishment of RBCs. The genomic region surrounding SNP s31152 has been under extreme selective pressure in domestic sheep [12]. The Ovine Hapmap SNP s31152 data (Table S8) was concordant with genotyping data in the three U.S. breeds reported here. The Rambouillet breed has a very low reference allele (allele A) frequency, 7.4% in the Hapmap data [12] and 8.9% in the Rambouillet population sampled here (Table S10). Three of the Polypay’s parent breeds were Finnsheep, Dorset and Rambouillet. The Polypay’s reference allele frequency is 34.2%, which falls between 85.4% in Finnsheep, 52.4% in Dorset, and 7.4% in Rambouillet. From the 74 Hapmap breeds, there are three different sheep breed clades with a very high instance of the reference allele: African origin, Northern European origin and high altitude (such as Tibetan) origin (Table S8). We hypothesize that the historical selective pressure may be related to anemia, which might be most severe at high altitude or under other specific environmental conditions. Both high altitude and certain types of parasitic infections can lead to anemia, which is often associated with RBC abnormalities [42].

To test this hypothesis, genotypes from the divergent artiodactyl *MYADM*-like repeat were analyzed for additional association with production traits in an expanded population of domestic sheep. The divergent artiodactyl *MYADM*-like repeat was associated with kilograms of lamb weaned (*P* = 2×10^−4^), with an increase of 15.5 kg of lambs weaned in a ewes lifetime in the alternate allele homozygotes (Table 4). Additionally, an association with ewe fourth year udder score (*P* = 0.024) was observed in the expanded production trait population (Table 4). The same alternate allele homozygotes showed diminished or less healthy udder condition (increased udder scores). While increased udder size is generally correlated with increased milk production and thus increased lamb weights, a larger udder may lead to an increased chance of structural failure, due to more tissue available to be damaged. Additionally, increased milk production can lead to increased occurrence of mastitis and potential death of mammary tissue. Further work is required to validate these associations with sheep production traits and continue testing the energy usage hypothesis for this RBC phenotype.

In addition to the divergent artiodactyl *MYADM*-like repeat region, the GWAS also identified 4 other genome-wide significant SNPs associated with erythrocyte traits. OAR1_19290808, s19887, s63011, and s48861 were associated with alteration to MCH, MCV, and Platelet count (Table S4-6). Of these, SNP s48861 is located on chromosome 20 near cyclin D3 (CCND3), bystin-like (BYSL), and ubiquitin specific peptidase genes (USP49). This region has been implicated in affecting hematological traits in human RBCs [3], [24]. It has been found that genes in the BYSL-CCND3 region are up-regulated in hematopoietic cells and have roles in hematopoiesis [24]. The bystin protein is a target of the c-MYC transcription factor, and it’s product plays a role in regulating protein synthesis in growing cells [43]. Additionally, CCND3 knockout mice show increased prevalence of heart abnormalities and severe anemia [44]. Further work is required to identify functional mutations underlying these GWAS results in sheep and to distinguish any regions which might show false positive association.

In conclusion, the GWAS identified a genomic region containing a divergent artiodactyl *MYADM*-like repeat associated with RBC phenotypes and sheep production traits. To our knowledge, no member of the *MYADM* gene family has been identified in relation to alterations in RBC morphology in any mammalian system. Thus, these results suggest *MYADM* family members as potential candidate genes for unexplained RBC morphology. Further, the associations of the divergent artiodactyl *MYADM*-like repeat with RBC and sheep production traits may help clarify potential reasons for the strong historical selection in the recent evolutionary history of domestic sheep [12]. Finally, additional genome-wide significant results identify multiple genomic regions for further investigation.

## Supporting Information

Figure S1 **Quantile-Quantile plot for Mean Corpuscular Hemoglobin Concentration (MCHC) GWAS.** Quantile-quanile plot for association with MCHC, where the red line indicates the expected distribution. The all-breeds, genotypic analysis is shown. Results show deviation from the expected distribution indicating population stratification unaccounted for in the analytic model.
(PDF)

Table S1 **Table S1**.
(PDF)

Table S2 **Table S2**.
(PDF)

Table S3 **Table S3**.
(PDF)

Table S4 **Table S4**.
(PDF)

Table S5 **Table S5**.
(PDF)

Table S6 **Table S6**.
(PDF)

Table S7 **Table S7**.
(PDF)

Table S8 **Table S8**.
(PDF)

Table S9 **Table S9**.
(PDF)

Table S10 **Table S10**.
(PDF)

Table S11 **Table S11**.
(PDF)

Table S12 **Table S12**.
(PDF)
