# Supplementary figures and images for: A Divergent Artiodactyl MYADM-like Repeat Is Associated with Erythrocyte Traits and Weight of Lamb Weaned in Domestic Sheep
Source: PLoS One. 2013 Aug 30;8(8):e74700. doi: 10.1371/journal.pone.0074700 (PMC3758307; doi:10.1371/journal.pone.0074700)

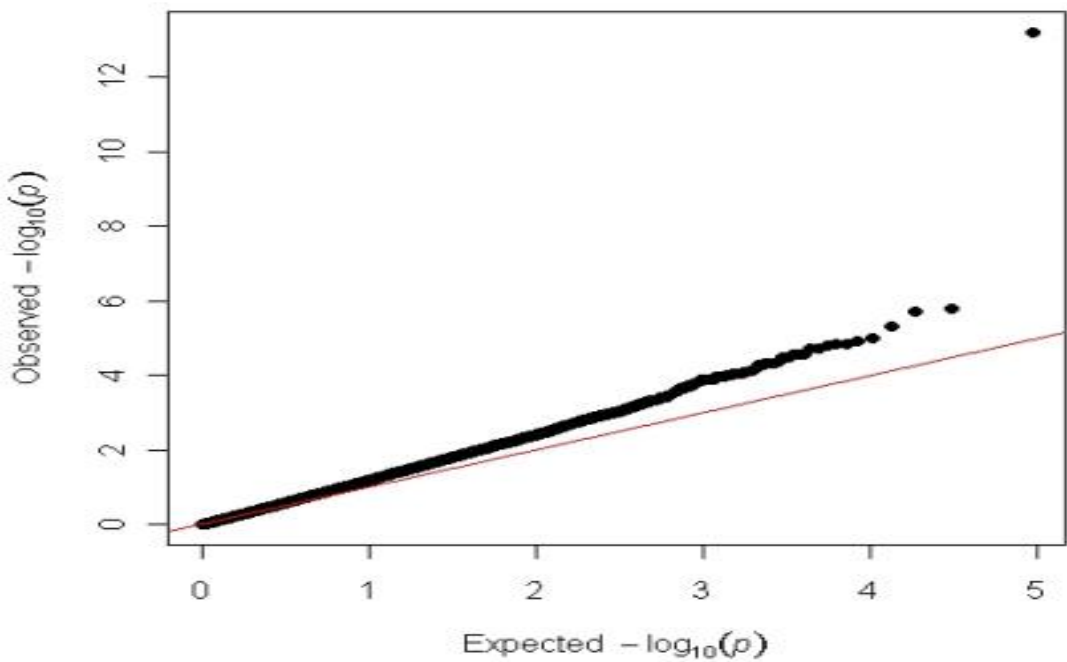

Supplement: Figure S1 — Quantile-Quantile plot for Mean Corpuscular Hemoglobin Concentration (MCHC) GWAS. Quantile-quanile plot for association with MCHC, where the red line indicates the expected distribution. The all-breeds, genotypic analysis is shown. Results show deviation from the expected distribution indicating population stratification unaccounted for in the analytic model. (PDF) [file pone.0074700.s001.pdf]
