# Supplementary material for: A Divergent Artiodactyl MYADM-like Repeat Is Associated with Erythrocyte Traits and Weight of Lamb Weaned in Domestic Sheep
Source: PLoS One. 2013 Aug 30;8(8):e74700. doi: 10.1371/journal.pone.0074700 (PMC3758307; doi:10.1371/journal.pone.0074700)
Supplement: Table S2 — (PDF) [file pone.0074700.s003.pdf]

Table S2: Genomic regions associated with Hermatocrit (HCT)

SNP	*Chr*	*Position (bp)*	*Best fitting model*	*Nominal P-value*	*Effect Size*	*Other Significant Phenotypes*	*Genes within 100 kb on either side*
s07047	20	46,575,517	genotypic	$6.5x10^{-6}$	2.760	None	*BMP6, TXNDC5*
