# Supplementary material for: A Divergent Artiodactyl MYADM-like Repeat Is Associated with Erythrocyte Traits and Weight of Lamb Weaned in Domestic Sheep
Source: PLoS One. 2013 Aug 30;8(8):e74700. doi: 10.1371/journal.pone.0074700 (PMC3758307; doi:10.1371/journal.pone.0074700)
Supplement: Table S3 — (PDF) [file pone.0074700.s004.pdf]

Table S3: Genomic regions associated with Hemoglobin (HGB)

SNP	*Chr*	*Position (bp)*	*Best fitting model*	*Nominal P-value*	*Effect Size*	*Other Significant Phenotypes*	*Genes within 100 kb on either side*
OAR15_23032896	15	22,075,369	recessive	3.2×10^{-6}	0.936	None	*C15H11orf34, BCO2*
OAR3_93074872	3	87,893,034	dominant	9.4×10^{-6}	0.496	None	None
