# Supplementary material for: A Divergent Artiodactyl MYADM-like Repeat Is Associated with Erythrocyte Traits and Weight of Lamb Weaned in Domestic Sheep
Source: PLoS One. 2013 Aug 30;8(8):e74700. doi: 10.1371/journal.pone.0074700 (PMC3758307; doi:10.1371/journal.pone.0074700)
Supplement: Table S4 — (PDF) [file pone.0074700.s005.pdf]

Table S4: Genomic regions associated with Mean Corpuscular Hemoglobin (MCH)

SNP	*Chr*	*Position (bp)*	*Best fitting model*	*Nominal P-value*	*Effect Size*	*Other Significant Phenotypes*	*Genes within 100 kb on either side*
**OAR1_192908082**	**1**	**178,924,433**	**recessive**	**$1.1x10^{-8}$**	**3.613**	**MCV, Platelets**	***LSAMP***
**s19887**	**2**	**247,548,978**	**recessive**	**$1.1x10^{-8}$**	**3.580**	**MCV, Platelets**	**None**
**s63011**	**11**	**11,443,436**	**recessive**	**$1.1x10^{-8}$**	**3.656**	**MCV, Platelets**	***BCAS3***
**s48861**	**20**	**15,785,304**	**recessive**	**$1.1x10^{-8}$**	**3.706**	**MCV, Platelets**	***PRICKLE4, TOMM6, CCND3, USP49, BYSL***
s36928	4	43,460,414	allelic	$1.1x10^{-6}$	0.869	MCV	*PHTF2, TMEM60, RSBN1L*
OAR4_45783408	4	43,394,799	genotypic	$6.7x10^{-7}$	0.862	MCV	*PHTF2, MAGI2, TMEM60*
s58817	1	262,343,186	recessive	$3.8x10^{-6}$	1.657	None	*C1H21orf33, ICOSLG, DNMT3L, AIRE, PFKL*
OAR10_80290780	10	73,373,701	allelic	$1.9x10^{-6}$	0.385	None	*HS6ST3*
s46674	16	62,860,047	genotypic	$2.4x10^{-6}$	0.452	None	*FAM173B, CMBL, CCT5*
s70539	1	38,713,856	dominant	$5.9x10^{-6}$	0.430	None	*PGM1, ROR1*
OAR3_55690836	3	52,646,098	dominant	$2.8x10^{-7}$	0.371	MCV	None
OAR2_168597479	2	159,122,199	allelic	$4.4x10^{-6}$	0.525	None	*LYPD6B*
OAR8_74319776	8	69,295,612	allelic	$8.0x10^{-6}$	0.169	None	None
