# Supplementary material for: A Divergent Artiodactyl MYADM-like Repeat Is Associated with Erythrocyte Traits and Weight of Lamb Weaned in Domestic Sheep
Source: PLoS One. 2013 Aug 30;8(8):e74700. doi: 10.1371/journal.pone.0074700 (PMC3758307; doi:10.1371/journal.pone.0074700)
Supplement: Table S5 — (PDF) [file pone.0074700.s006.pdf]

Table S5: Genomic regions associated with Mean Corpuscular Volume (MCV)

SNP	*Chr*	*Position (bp)*	*Best fitting model*	*Nominal P-value*	*Effect Size*	*Other Significant Phenotypes*	*Genes within 100 kb on either side*
**s19887**	**2**	**247,548,978**	**genotypic**	**8.9×10^{-10}**	**11.678**	**MCH, Platelets**	**None**
**s48861**	**20**	**15,785,304**	**recessive**	**1.1×10^{-9}**	**11.947**	**MCH, Platelets**	***CCND3, PRICKLE4, USP49, TOMM6, BYSL***
**OAR1_192908082**	**1**	**178,924,433**	**recessive**	**1.1×10^{-9}**	**11.916**	**MCH, Platelets**	***LSAMP***
**s63011**	**11**	**11,443,436**	**recessive**	**1.1×10^{-9}**	**45.963**	**MCH, Platelets**	***BCAS3***
s32816	3	223,111,190	genotypic	1.6×10^{-7}	3.522	None	None
s27352	2	66,226,023	recessive	3.2×10^{-6}	6.570	None	*C8H9orf135*
s19950	3	137,737,526	allelic	7.7×10^{-7}	1.236	None	*OR8S1, C12orf54*
**s31152**	**18**	**19,342,316**	**allelic**	**2.5×10^{-6}**	**2.165**	**MCHC**	***MYADM-like***
s09255	4	104,405,037	genotypic	2.6×10^{-6}	1.618	None	None
s14642	7	52,043,688	genotypic	2.4×10^{-6}	1.117	None	*CCPG1, RSL24D1, RAB27A, MIR628, PIGB*
OAR2_83377214	2	78,148,767	allelic	6.1×10^{-6}	1.075	None	None
OAR3_55690836	3	52,646,098	dominant	1.3×10^{-6}	0.979	MCH	None
OAR4_45783408	4	43,394,799	genotypic	4.2×10^{-6}	2.441	MCH	*PHTF2, MAGI2, TMEM60*
s36928	4	43,460,414	genotypic	4.7×10^{-6}	0.869	MCH	*PHTF2, TMEM60, RSBN1L*
s23722	3	178,956,951	genotypic	5.6×10^{-6}	2.490	None	*MB, APOL6, RBFOX2*
OAR3_68338269	3	64,685,999	dominant	1.8×10^{-6}	0.895	None	None
OAR13_22065360	13	19,670,268	dominant	3.4×10^{-6}	0.881	None	None
OAR4_24030182	4	22,932,880	dominant	5.7×10^{-6}	0.824	None	*DGKB*

OAR4_24543945_X	4	23,435,546	dominant	$7.8x10^{-6}$	0.854	None	None
OAR26_5062133	26	4,467,736	dominant	$9.2x10^{-6}$	0.809	None	None
