# Supplementary material for: A Divergent Artiodactyl MYADM-like Repeat Is Associated with Erythrocyte Traits and Weight of Lamb Weaned in Domestic Sheep
Source: PLoS One. 2013 Aug 30;8(8):e74700. doi: 10.1371/journal.pone.0074700 (PMC3758307; doi:10.1371/journal.pone.0074700)
Supplement: Table S6 — (PDF) [file pone.0074700.s007.pdf]

Table S6: Genomic regions associated with Platelet Count

SNP	*Chr*	*Position (bp)*	*Best fitting model*	*Nominal P-value*	*Effect Size*	*Other Significant Phenotypes*	*Genes within 100 kb on either side*
**OAR1_192908082**	**1**	**178,924,433**	**recessive**	**1.3×10^{-6}**	**923.247**	**MCV, MCH**	***LSAMP***
**s19887**	**2**	**247,548,978**	**recessive**	**1.3×10^{-6}**	**972.246**	**MCV, MCH**	**None**
**s63011**	**11**	**11,443,436**	**recessive**	**1.3×10^{-6}**	**947.649**	**MCV, MCH**	***BCAS3***
**s48861**	**20**	**15,785,304**	**recessive**	**1.3×10^{-6}**	**919.122**	**MCV, MCH**	***CCND3, PRICKLE4, USP49, TOMM6, BYSL***
OAR26_17762928	26	14,878,641	allelic	1.8×10^{-6}	115.434	None	*TLR3, FAM149A*
s30011	18	34,804,342	allelic	2.0×10^{-6}	111.923	None	*TGIF1*
OAR12_75421182	12	68,648,670	allelic	2.8×10^{-6}	113.556	None	*VASH2, RPS6KC1, ANGEL2*
OAR11_39358742	11	36,917,331	allelic	4.3×10^{-6}	104.244	None	*ZNF652, PHOSPHO1, ABI3, GNGT2, B4GALNT2*
s63282	15	51,301,670	allelic	5.5×10^{-6}	111.754	None	*PLEKHB1, RAB6A, WTH3DI*
OAR3_85156224	3	80,592,907	allelic	6.2×10^{-6}	144.603	None	*THADA, PLEKHH2*
OAR11_39405168	11	36,968,763	genotypic	6.2×10^{-6}	105.675	None	*PHOSPHO1, ABI3, GNGT2, B4GALNT2, IGF2BP1*
OAR11_39377571	11	36,938,798	allelic	7.0×10^{-6}	102.007	None	*ZNF652, PHOSPHO1, ABI3, GNGT2, B4GALNT2*
s31218	8	3,433,729	genotypic	9.7×10^{-6}	484.713	None	None
s24871	25	27,201,713	dominant	1.8×10^{-7}	96.878	None	*UNC5B*
OAR2_58331140	2	54,213,818	dominant	1.2×10^{-6}	87.041	None	None
s21480	11	43,893,157	dominant	4.7×10^{-6}	80.119	None	*GJC1, CCDC43, HIGD1B, RPL39L, DBF4B, ADAM11, EFTUD2,*

							C19H17orf104
OAR1_214792719	1	198,996,411	dominant	$7.4x10^{-6}$	93.781	None	None
