# Supplementary material for: A Divergent Artiodactyl MYADM-like Repeat Is Associated with Erythrocyte Traits and Weight of Lamb Weaned in Domestic Sheep
Source: PLoS One. 2013 Aug 30;8(8):e74700. doi: 10.1371/journal.pone.0074700 (PMC3758307; doi:10.1371/journal.pone.0074700)
Supplement: Table S7 — (PDF) [file pone.0074700.s008.pdf]

Table S7: Significant Phenotypic Values for s31152 by Genotype

	AA	AG	GG	Nominal P-value
MCHC (%)	36.252	34.402	33.991	6.2×10^{-14}
MCV (fL)	32.398	33.923	34.563	2.5×10^{-6}
HCT (%)	30.992	33.213	33.709	0.0015
