# Supplementary material for: A Divergent Artiodactyl MYADM-like Repeat Is Associated with Erythrocyte Traits and Weight of Lamb Weaned in Domestic Sheep
Source: PLoS One. 2013 Aug 30;8(8):e74700. doi: 10.1371/journal.pone.0074700 (PMC3758307; doi:10.1371/journal.pone.0074700)
Supplement: Table S8 — (PDF) [file pone.0074700.s009.pdf]

Table S8: Genotypic Frequency of s31152 marker in 74 Ovine Hapmap Breeds

Breed	Abbrev	n	AA	AG	GG	Ref allele frequency	Origin
MacarthurMerino	MCM	10	0	0	10	0.000	SW Europe
Sakiz	SKZ	22	0	0	22	0.000	SW Asia
SardinianAncestralBlack	SAB	20	0	0	20	0.000	SW Europe
AustralianMerino	MER	50	0	2	48	0.020	SW Europe
Altamurana	ALT	24	0	1	23	0.021	SW Europe
Ojalada	OJA	24	0	1	23	0.021	SW Europe
Rasaaragonesa	RAA	22	0	1	21	0.023	SW Europe
Churra	CHU	120	0	7	113	0.029	SW Europe
IndianGarole	GAR	26	1	0	25	0.038	Asia
AustralianPollMerino	APM	98	0	8	90	0.041	SW Europe
AustralianIndustryMerino	MER	88	1	9	78	0.063	SW Europe
Castellana	CAS	23	0	3	20	0.065	SW Europe
Rambouillet	RMB	102	0	15	87	0.074	SW Europe
Leccese	LEC	24	0	5	19	0.104	SW Europe
Chios	CHI	23	1	3	19	0.109	SW Europe
Wiltshire	WIL	23	0	5	18	0.109	Northern Europe
MeatLacaune	LAC	78	3	14	61	0.128	SW Europe
Merinolandschaf	MLA	24	0	7	17	0.146	SW Europe
BorderLeicester	BRL	48	2	12	34	0.167	Northern Europe
Comisana	COM	24	0	10	14	0.208	SW Europe
MilkLacaune	LAC	103	6	35	62	0.228	SW Europe
ChineseMerino	CME	23	1	9	13	0.239	SW Europe
AustralianPollDorset	APD	108	7	39	62	0.245	Northern Europe
BlackHeadedMountain	BHM	24	2	8	14	0.250	Central Europe
NewZealandRomney	ROM	24	1	10	13	0.250	Northern Europe
EastFriesianBrown	EFB	39	5	11	23	0.269	Central Europe
IrishSuffolk	ISF	55	4	25	26	0.300	Northern Europe
EastFriesianWhite	EFW	9	1	4	4	0.333	Central Europe
SwissWhiteAlpineSheep	SWA	24	3	10	11	0.333	Central Europe
BrazilianCreole	BCS	23	3	11	9	0.370	Americas
CyprusFatTail	CFT	30	5	13	12	0.383	SW Asia
GermanTexel	GTX	46	4	28	14	0.391	Northern Europe
Boreray	BOR	17	3	8	6	0.412	Northern Europe
AustralianCoopworth	CPW	19	2	12	5	0.421	Northern Europe
AustralianSuffolk	ASU	109	22	48	39	0.422	Northern Europe
Galway	GAL	49	7	28	14	0.429	Northern Europe
BangladeshiBGE	BGE	24	5	11	8	0.438	Asia
GulfCoastNative	GCN	94	23	42	29	0.468	Americas
ScottishTexel	STX	80	18	40	22	0.475	Northern Europe

Moghani	MOG	34	10	13	11	0.485	SW Asia
BangladeshiGarole	BGA	24	6	12	6	0.500	Asia
Afshari	AFS	37	10	18	9	0.514	SW Asia
NewZealandTexel	NTX	24	6	13	5	0.521	Northern Europe
DorsetHorn	DSH	21	4	14	3	0.524	Northern Europe
SwissBlack-BrownMountainSheep	SBS	24	8	10	6	0.542	Central Europe
Deccani	IDC	24	9	9	6	0.563	Asia
Sumatra	SUM	24	6	15	3	0.563	Asia
SwissMirrorSheep	SMS	24	9	11	4	0.604	Central Europe
AfricanDorper	ADP	21	10	9	2	0.690	Africa
Soay	SOA	110	56	44	10	0.709	Northern Europe
EngadineRedSheep	ERS	24	12	11	1	0.729	Central Europe
SantaInes	BSI	47	25	19	3	0.734	Americas
BarbadosBlackBelly	BBB	24	13	10	1	0.750	Americas
BundnerOberlanderSheep	BOS	24	13	10	1	0.750	Central Europe
MoradaNova	BMN	22	12	9	1	0.750	Americas
StElizabeth	STE	10	5	5	0	0.750	Americas
Karakas	KRS	18	10	8	0	0.778	SW Asia
ScottishBlackface	SBF	56	36	18	2	0.804	Northern Europe
Qezel	QEZ	35	24	9	2	0.814	SW Asia
Garut	GUR	22	16	5	1	0.841	Asia
Finnsheep	FIN	99	74	21	4	0.854	Northern Europe
RedMaasai	RMA	45	35	8	2	0.867	Africa
Norduz	NDZ	20	15	5	0	0.875	SW Asia
ValaisBlacknoseSheep	VBS	24	18	6	0	0.875	Central Europe
AfricanWhiteDorper	AWD	6	5	1	0	0.917	Africa
ValaisRedSheep	VRS	24	20	4	0	0.917	Central Europe
EthiopianMenz	EMZ	34	29	5	0	0.926	Africa
OldNorwegianspaelsau	NSP	15	13	2	0	0.933	Northern Europe
Changthangi	CHA	29	27	2	0	0.966	Asia
RonderibAfrikaner	RDA	17	16	1	0	0.971	Africa
NamaquaAfrikaner	NQA	12	12	0	0	1.000	Africa
Spael-coloured	NSP	3	3	0	0	1.000	Northern Europe
Spael-white	NSP	32	32	0	0	1.000	Northern Europe
Tibetan	TIB	37	37	0	0	1.000	Asia
