# Supplementary material for: A Divergent Artiodactyl MYADM-like Repeat Is Associated with Erythrocyte Traits and Weight of Lamb Weaned in Domestic Sheep
Source: PLoS One. 2013 Aug 30;8(8):e74700. doi: 10.1371/journal.pone.0074700 (PMC3758307; doi:10.1371/journal.pone.0074700)
Supplement: Table S9 — (PDF) [file pone.0074700.s010.pdf]

Table S9: Genotypic Frequency of s31152 marker in RBC GWAS Population

	Columbia	Polypay	Rambouillet
AA	0	18	2
AG	3	93	45
GG	64	84	200
