# Supplementary material for: A Divergent Artiodactyl MYADM-like Repeat Is Associated with Erythrocyte Traits and Weight of Lamb Weaned in Domestic Sheep
Source: PLoS One. 2013 Aug 30;8(8):e74700. doi: 10.1371/journal.pone.0074700 (PMC3758307; doi:10.1371/journal.pone.0074700)
Supplement: Table S11 — (PDF) [file pone.0074700.s012.pdf]

Table S11: Failed Primer Sets in *MYADML* Alternate Allele

v.3.1 Start	*v. 3.1 Stop*	*Primer Length*	*Sequence (5' – 3')*	*Amplicon Size*
**19271842**	**19271864**	**23**	**TGCAAGACTGGTTCACCCCAGAT**	**330**
**19272149**	**19272172**	**24**	**CCGGCTTTATGTCAGTGATACGTG**	
19275129	19275152	24	GTCTTGAGCAGGGATGGAGAATGG	362
19275468	19275491	24	AGACTTGGCCTCAGACCCAAAAGG	
19276549	19276572	24	ATTCTCTGCACAGTCCAGTGGCCT	280
19276806	19276829	24	CATTCCAGGGTGCTAGGCATCTGA	
19278758	19278781	24	AGACTGGGGATTATAGAACTCTCC	555
19279290	19279313	24	ACTCTTTAGCACCGTTCTCACAGC	
19279291	19279313	23	CTGTGAGAACGGTGCTAAAGAGT	4916
19284184	19284207	24	GCTCATGCCCACGGCTTTGGTATA	
19279882	19279905	24	TTGCTTCTTCCCATATCCGAAGTC	4288
19284147	19284170	24	CCAACTTCATGGTCATGCCACCTT	
19280636	19280658	23	ACAAACTGCTACTGGCCTCAGTG	3908
19284522	19284544	23	TTGGGGTCCCCTGACCCAATGCA	
19280887	19280910	24	AAGGGGCTCCACGAAACCCAGGTT	3335
19284199	19284222	24	ACTGTAGAGCACAGAGCTCATGCC	
19281746	19281768	23	AAGGGGACGTGCATTGTACAGCC	1790
19283514	19283536	23	CTCCATAGATAATGGCCAGCTTA	
19281686	19281709	24	GGTCTCTGCTTCAAATTTCCTGCT	1644
19283307	19283330	24	ACAGAGCCCTGAGCCATTTGTAGG	
19281833	19281855	23	CAACTAAGTTACTCCCAGGGGAA	1549
19283360	19283382	23	CAGGATCTGAGGCCATTGGTTCC	
19282502	19282524	23	CACTGATGTAGAGGAGGACGAAG	589
19283069	19283091	23	TTGGAGAGGGGACGTAGGTAACT	
19282587	19282609	23	CAGATTCAGCAGGATGGTTACTG	453
19283018	19283040	23	CACTGTGACCCTATTCATAGCCA	
19283501	19283524	24	CTGTGGATCTCTCTAAGCTGGCCA	372
19283850	19283873	24	AGTCAAAAGGACACTCCATAGTCC	

19288062	19288085	24	AGGAGCAGGAATGTTCAGGAGGAG	466
19288505	19288528	24	GGCTGAATTCAGACACCATGGAAC	
19298161	19298184	24	CTGAGGGGAAGGTAGTGCAGGAAT	379
19298517	19298540	24	GGCTACCAGATCTTTCTGAATCTG	
19313211	19313234	24	GACCCTGTTATAGTTCTATGAGGC	370
19313558	19313581	24	CAATGCTGAAGTACTCAGAGCGGA	
19314883	19314906	24	TCCTCCTAGAGGAGGTGATTTAGG	251
19315111	19315134	24	TCTAACTCCTTACCTTGCACCACC	
19317198	19317221	24	AGATGCTTCCTGGATGAAAGGGTC	249
19317424	19317447	24	GCCTCAGTATATCTGCTGTCACTG	

*Bold denotes primer set used as AA allele-specific primer set
