# Supplementary material for: A Divergent Artiodactyl MYADM-like Repeat Is Associated with Erythrocyte Traits and Weight of Lamb Weaned in Domestic Sheep
Source: PLoS One. 2013 Aug 30;8(8):e74700. doi: 10.1371/journal.pone.0074700 (PMC3758307; doi:10.1371/journal.pone.0074700)
Supplement: Table S12 — (PDF) [file pone.0074700.s013.pdf]

Table S12: s31152 genotype contrasts for lifetime kilograms of lamb weaned (LkgLW) and udder condition at fourth year (UDDER)

	AA	AG	GG	P-value
LkgLW (in kg)	-	225.3	241.5	0.0002
UDDER	-	2.13	2.49	0.0335
